# Analysis of a Parent-Initiated Social Media Campaign for Hirschsprung’s Disease

**DOI:** 10.2196/jmir.3200

**Published:** 2014-12-11

**Authors:** Kristy Wittmeier, Cindy Holland, Kendall Hobbs-Murison, Elizabeth Crawford, Chad Beauchamp, Brodie Milne, Melanie Morris, Richard Keijzer

**Affiliations:** ^1^Centre for Healthcare InnovationUniversity of ManitobaWinnipeg Regional Health AuthorityWinnipeg, MBCanada; ^2^Department of Pediatrics and Child HealthUniversity of ManitobaWinnipeg, MBCanada; ^3^Manitoba Institute of Child HealthWinnipeg, MBCanada; ^4^Child Health ProgramHealth Sciences CentreWinnipeg, MBCanada; ^5^Department of SurgeryUniversity of ManitobaWinnipeg, MBCanada; ^6^Swish Productions Ltd.Winnipeg, MBCanada; ^7^Direct Focus Marketing Communications Inc.Winnipeg, MBCanada

**Keywords:** Hirschsprung’s Disease, social media, patient oriented research, knowledge translation

## Abstract

**Background:**

Social media can be particularly useful for patients or families affected by rare conditions by allowing individuals to form online communities across the world.

**Objective:**

Our aim in this study was to conduct a descriptive and quantitative analysis of the use of a social media community for Hirschsprung’s Disease (HD).

**Methods:**

In July 2011, a mother of a child with HD launched the “Shit Happens” campaign. The campaign uses social media (blogs, Twitter, and Facebook) to engage other families affected by HD. Internet analytics including Google Analytics and Facebook Insights were used to evaluate the reach and responsiveness of this campaign.

**Results:**

On the day the HD campaign was launched, 387 people viewed the blog “Roo’s Journey”. Blog views have now exceeded 5400 views from 37 countries. The Facebook page extends to 46 countries, has an average post reach of 298 users, 1414 “likes”, and an overall reach of 131,032 users. The campaign has 135 Twitter followers and 344 tweets at the time of writing. The most common question posted on the Facebook page is related to treatment for extreme diaper rash. Responsiveness assessment demonstrated that within 2 hours of posting, a question could receive 143 views and 20 responses, increasing to 30 responses after 5 hours.

**Conclusions:**

Social media networks are well suited to discussion, support, and advocacy for health-related conditions and can be especially important in connecting families affected by rare conditions. The HD campaign demonstrates the reach and responsiveness of a community that primarily relies on social media to connect families affected by HD. Although responsive, this community is currently lacking consistent access to evidence-based guidance for their common concerns. We will explore innovative consumer-researcher partnerships to offer a solution in future research.

## Introduction

Hirschsprung’s disease (HD) is a rare condition that is estimated to affect 1 in 5000 live births [1]. This disease primarily affects the innervation of the gastrointestinal tract due to failure in neural crest cell migration during the embryonic period. As a result, a section of bowel is left aganglionic, resulting in a portion of bowel essentially paralyzed and unable to mobilize stool [2].

HD typically presents in infancy with a delay or failure to pass meconium, ongoing difficulty passing stool, abdominal distension, and intolerance to feeding. However, HD may also present in early childhood or later with mild symptoms in association with growth concerns and chronic constipation. Treatment generally consists of a pull-through surgery, where the affected segment of bowel is removed and the healthy portion of bowel is re-anastomosed. Historically, there has been advancement from a three-step procedure to current practice of a single or two-step procedure with use of minimally invasive techniques when appropriate [3]. Researchers continue to investigate the possible use of cell transplantation in the antenatal period [3,4].

Despite promising advances in diagnostics and surgical techniques, there remain many challenges that individuals with HD and their caregivers encounter in the day-to-day management of this condition. In the postoperative period, challenges include irregular stool patterns, ongoing incontinence, impaired psychosocial function, the potential of enterocolitis, and concerns regarding quality of life for both the child and caregiver [4,5].

Specific to caregivers, it is becoming better recognized that raising a child with a rare congenital anomaly presents a number of unique challenges that can contribute to a higher level of parental stress [6]. Stress is associated with concerns regarding the competencies required to care for a child with medical needs, or feelings of social isolation [7,8]. This is exaggerated in large countries such as Canada and the United States where the closest pediatric hospital might be hundreds of kilometers away, making it difficult to obtain relevant advice or adequate support in a timely fashion. Traditional social support networks such as family, friends, and neighbors may be inadequate or ill equipped to understand and address the issues and questions specific to parenting a child with complex needs, which further aggravates parental stress.

The Internet is a common resource accessed by the general public on medical issues [9]. A recent report indicates that 59% of adult Internet users search for health information online [9]. Simply searching “Hirschsprung’s Disease” on Google returns over 660,000 results within 0.24 seconds, with the top hits containing detailed information on the disease definition, diagnosis, and surgical procedures used to manage the condition. Information to guide caregivers in day-to-day intricacies of the life of a child with HD as well as on longer-term complications of the condition is unfortunately less available to the general public.

Social media overcomes the limitations of more static websites designed primarily to disseminate information, by facilitating the development of relationships and communities around shared interests [10,11]. Social media provides a portal to quick, interactive communication and a venue for people with common interests, including health-related interests to connect [9], such as those affected by chronic and rare diseases [12-14]. Acknowledging that social media has changed the way people collect and disseminate information [15], the potential for social media to help improve disease management and health outcomes requires further study.

In an effort to build a visible and easily accessible interactive Web-based community for caregivers of children with HD, a parent within the HD community in Manitoba, Canada, has collaborated with a marketing team to develop, implement, and maintain an ongoing social media campaign, entitled “Shit Happens”. This mother (EC; “Liz”) has partnered with our research team to evaluate the campaign as part of a broader effort to understand and improve the care of children with HD nationally and internationally. The primary objective of this study was to perform a descriptive analysis of the HD social media campaign and to conduct a quantitative assessment of the reach and responsiveness of the community. A secondary objective was to conduct a preliminary content analysis of priority areas for health information needs as identified by HD social media community members.

## Methods

### Overview

The targeted audience of this campaign was caregivers of children with HD and those living with HD that self-engaged in social media. Health care professionals were not a targeted group at this time. The campaign has facilitated the development of a virtual community, defined as “online social networks in which people with common interests, goals, or practices interact to share information and knowledge, and engage in social interactions” [16]. The community and their interactions were each analyzed through a combination of embedded performance measures and available Internet analysis tools on the blog, Facebook, and Twitter. Collectively, these methods provide data to assess the reach and responsiveness of the HD social media campaign, indicators of community engagement, and campaign impact.

### Reach

“The Roo’s Journey” blog was analyzed using Google Analytics allowing real-time and custom data reporting through the development of an indicator dashboard. Both dimensions (describe data) and metrics (measure data) were built in to provide a custom report [17]. Specific items monitored on the dashboard for Roo’s Journey include new visits, unique visitors, visits by (Internet) browser, mapping (geographical representation of visits), average visit duration, and pages per visit. Facebook’s Insights dashboard provides data on the number of countries the account has reached, age category and gender of users, potential reach (includes friends of page fans), “likes”, post reach (number of unique people who saw that individual post), and feedback. Past data provided include number of active monthly users and total post views. Twitter analytics include the number of followers and number of tweets.

### Responsiveness

Community responsiveness was assessed by quantifying the volume and timing of feedback for a question posted to Facebook by the site administrator. Priority areas for information needs were determined through a qualitative analysis and identification of recurring themes within questions posed by the Facebook community members. As the social media platforms were within the public domain and the data were collected by the site administrator, the University of Manitoba Research Ethics Office waived the need for ethical review of this study.

## Results

### Overview

**Figure 1:**
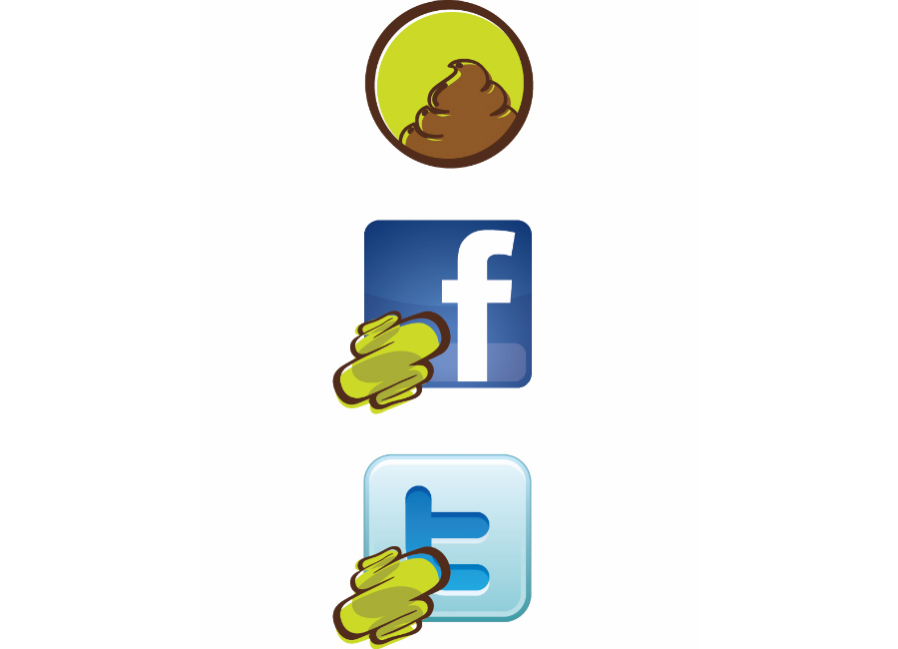
Blog, Facebook, and Twitter logos for HD campaign.

In July 2011, the mother of a 2-year old with HD, launched the “Shit Happens” campaign (Figure 1).

This campaign included a blog titled Roo’s Journey, describing a family’s experience with HD, a Facebook page (Hirschsprung’s Community), and a Twitter account (@shithapps). The collective objective of these three forums was to support families living with HD and raise awareness of the disease. The HD campaign name “Shit Happens” was chosen from a marketing perspective to create a brand that would stand out to enhance campaign uptake and spread. The campaign name was also chosen to resonate with potential community members and break the barriers of an otherwise socially awkward topic. In Liz’s words:

Many children with HD suffer from chronic constipation and occasional bowel obstruction. For a child and family with HD the idea of “shit” happening tends to bring feelings of joy and relief. When a bowel movement finally occurs it is not for the faint of heart…hence the name (Shit Happens). Laugh or cry…may as well laugh!

The campaign creator is the primary contributor and chief administrator of all three social media platforms. The Hirschsprung’s community on Facebook has daily interaction as questions and concerns arise. While the Facebook page was created to engage caregivers, its primary function was to create a support network. Online and offline supportive relationships and networks have developed through introductions and referrals. Facebook posts were shared as tweets through Twitter.

**Figure 2:**
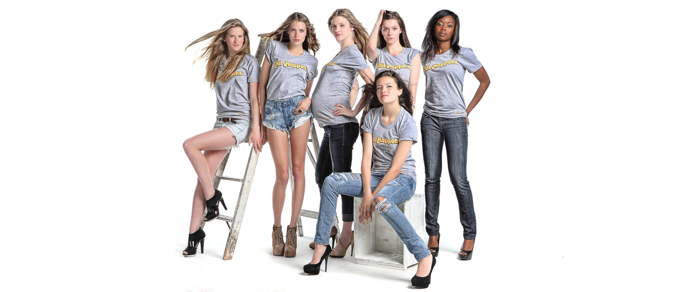
Image from HD Around the Globe campaign featuring models wearing the campaign T-shirt.

The Roo’s Journey blog provides an in-depth and honest portrayal of caring for a child with HD. Facebook and Twitter are used to share links to Roo’s Journey, other blogs, videos, and information on conferences and events that may be of interest to the HD community. In 2012, the HD Around the Globe campaign was launched within the HD Community using all social media platforms to obtain wider recognition of the disease. This ongoing campaign features photos of individuals from the HD community, celebrities, and models wearing the HD Community “SHIT HAPPENS” T-shirt (Figure 2).

To ensure the sites are efficiently managed, the posts are monitored daily. All Facebook posts are screened prior to reposting, and posts that are considered inappropriate or potentially harmful are immediately deleted. Since inception, there have been only two post deletions; these were due to misuse of language related to expression of frustration. All posts and comments to these sites can be viewed publicly, while personal messaging or email to the creator in the case of the blog remain private. For Facebook and Twitter, participating users must agree to the terms and conditions outlined by these social media platforms. HD community participation requires no additional inclusion criteria, although it is noted that the groups were created as a resource to those affected by HD. Outside of the social media realm, this campaign extends promotion of the Hirschsprung’s Community through events such as fashion events in shopping centres, Fashion Rocks (an annual fundraiser), and Easton’s memorial poker run (Nebraska, USA). The campaign creator’s personal public relations company sustains the campaign in collaboration with an affiliated marketing company (Swish Productions Ltd. and Direct Focus Marketing Communications Inc.). Local and international health care professionals with expertise in HD demonstrate campaign support with presentations and participation at HD fundraising opportunities and by informing families whom they interact with about this ongoing campaign.

**Figure 3:**
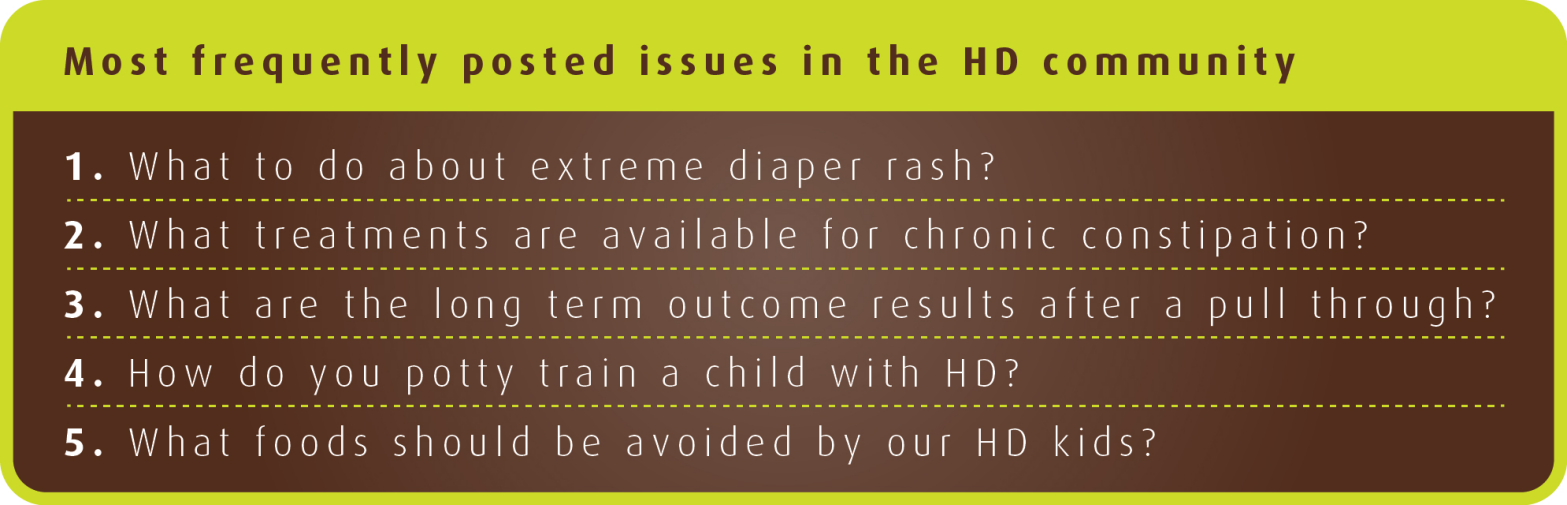
The five most common issues raised in HD community on the Facebook page.

The most common question posted on the HD Facebook page is regarding the management of severe diaper rash, followed closely by questions regarding bowel treatments, long-term outcomes, potty training, and dietary issues (Figure 3).

### Reach

On the day of the launch of the HD Community Campaign, 387 people viewed the Roo’s Journey blog. Almost 3 years post-launch, there have been 5487 blog views and/or visitors, encompassing 37 countries. The blog has been most often read in the United States, Canada, and the United Kingdom.

**Figure 4:**
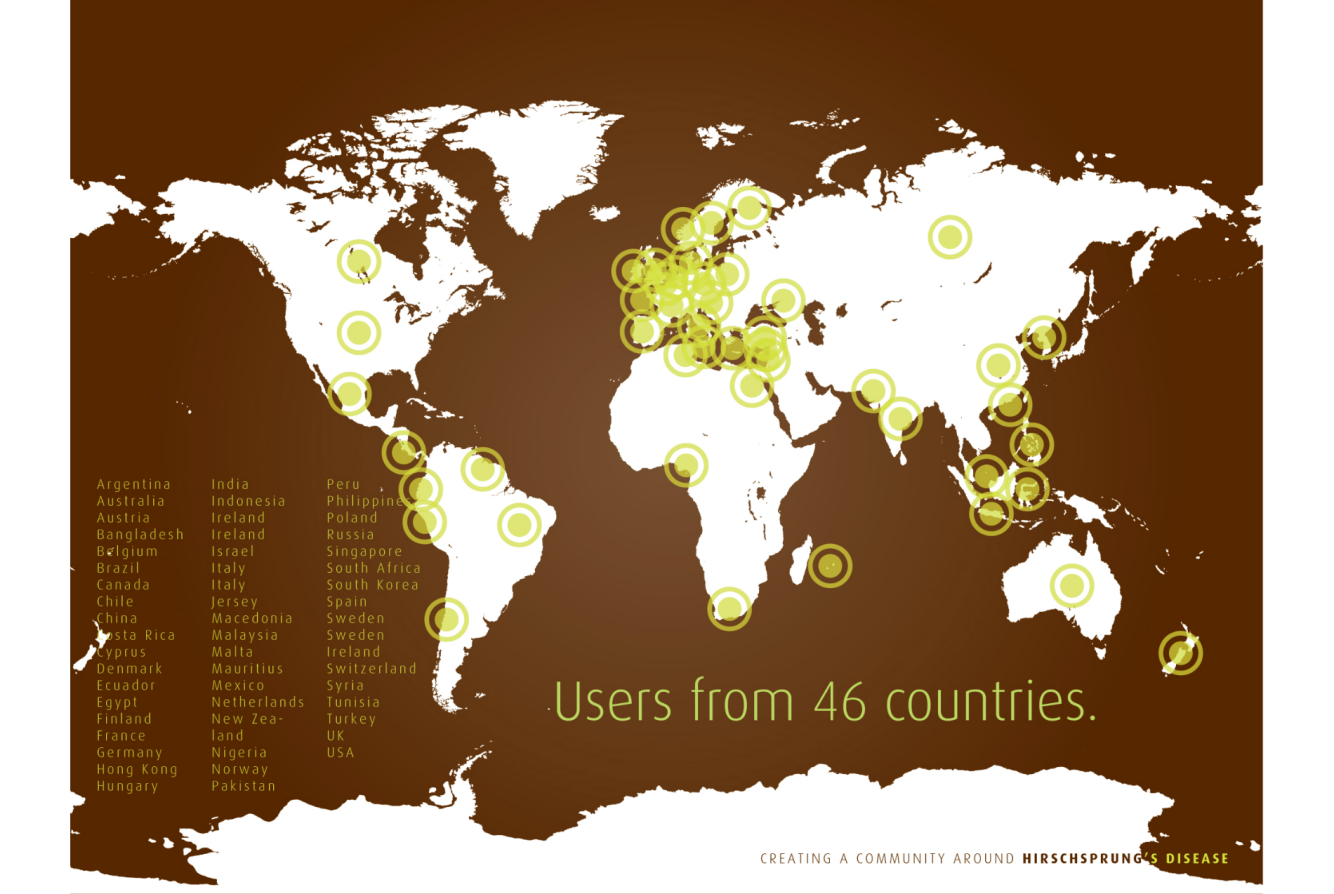
Reach of HD Community Facebook page.

The Facebook page extends to 46 countries with the average user being female between the ages of 25-44 years (Figure 4). Past metrics provided by Facebook Insights indicate that there were 1130 monthly users of the HD Community Facebook page. At the time of writing, there were a total of 1414 “likes”,and based on the last 28 days, the average post reach (number of unique people who viewed the post) was 298 people, with a total overall reach of the community (includes Facebook page users and their friends) of 131,032 people.

On Twitter there are currently 135 followers, and 344 tweets have been posted.

### Responsiveness

**Figure 5:**
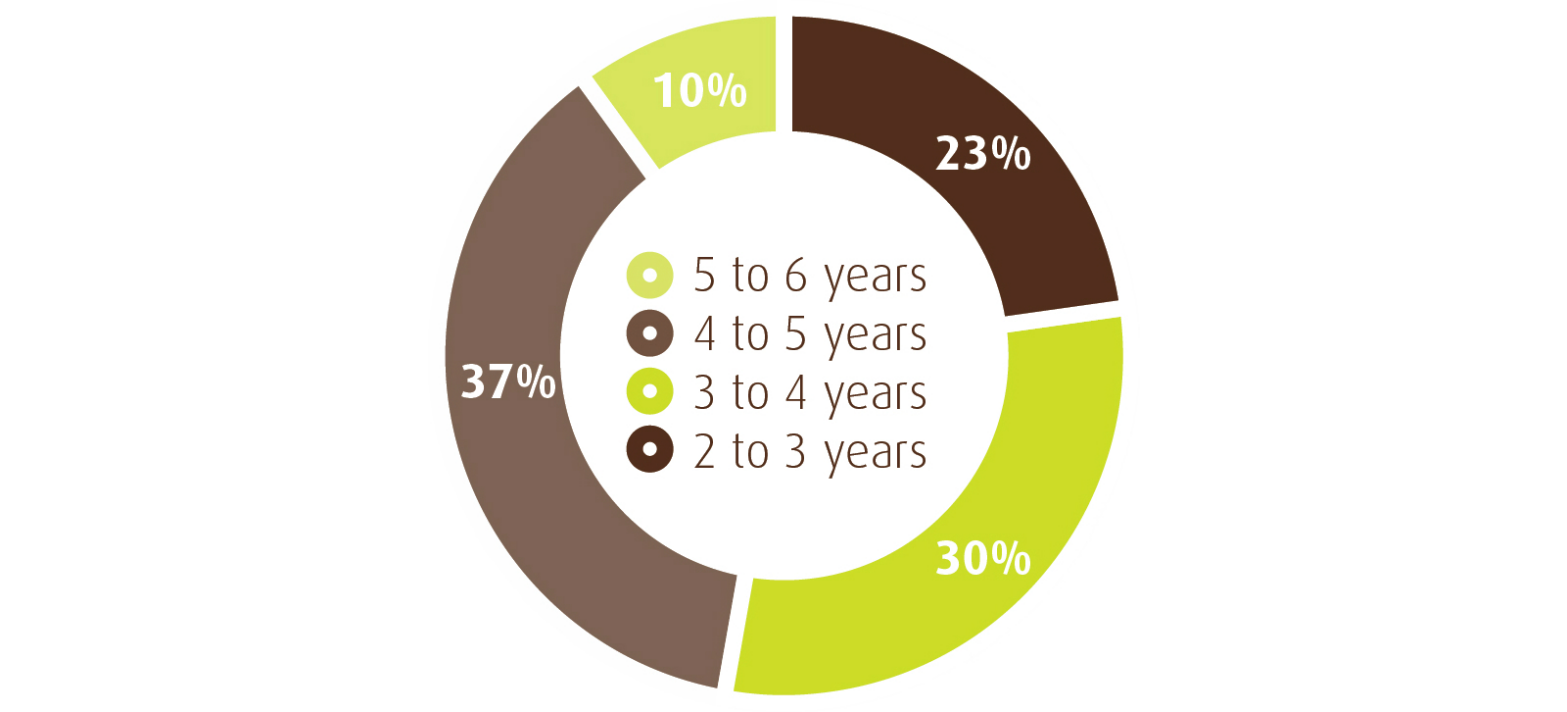
Reported percentages of “age of potty training” in response to the question “At what age was your child potty trained?”.

Community responsiveness was measured with a post asking, “At what age was your child potty trained?”. The question was viewed 143 times within 2 hours, and 20 individuals provided responses (14% of viewers). Five hours after the question was posed, 30 responses were posted (Figure5) with the majority indicating that despite being potty trained, their children still experienced ongoing incontinence issues.

Similarly, when a question was posted regarding management of diaper rash and diarrhea after pull-through surgery, 413 people viewed the post within 2 hours and 20 members (5% of viewers) replied offering advice and support on how to deal with this issue based on their experience.

## Discussion

### Principal Findings

Social media can be a powerful tool to create a community for caregivers of children born with rare diseases such as HD, connecting individuals across the world within minutes. Demonstrating reach of a large geography and many participants, this caregiver community is engaged and responsive. A content analysis of Facebook postings highlights current priority areas for knowledge and resources among caregivers within this community.

From a health research perspective, this community provides the ideal opportunity for integrated knowledge translation by providing a venue for interaction between researchers and knowledge users [18]. A caregiver identified a gap in available resources for parents and has created a community to address this gap. Community members now use the social media platforms as information sharing tools, to exchange knowledge, advice, and experiences. What has been notably absent however, is evidence-informed input from health care providers—a trend observed in previous evaluation of health-related social media forums [19]. In this age of evidence-based practice and knowledge translation, it is necessary to consider the role of the clinician and the researcher within the social media sphere. Now, through this new partnership between caregivers, academics, and clinicians, the HD community is well poised to systematically identify and address knowledge gaps with evidence-based tools and resources.

Moving forward, a detailed analysis of issues raised within the HD community will provide the basis of a needs assessment to inform new research including knowledge synthesis and translation activities. Previous research examining Twitter and smoking cessation has highlighted inconsistencies in advice or tweet content relative to existing treatment guidelines [19]. A researcher-knowledge user partnership can help overcome the risk of sharing misinformation associated with consumers seeking health care advice via the Internet.

Guidelines and ethics documents are becoming more widely available for clinicians and researchers who wish to engage in social media [15,20-23]. These should be reviewed and adhered to appropriately. Basic considerations include respecting patient confidentiality, maintaining appropriate boundaries, disclosure of competing or conflicting interests, and consideration of professional reputation [20-23]. The same standards apply to patient-caregiver interactions in person and online, and if respected, can safely broaden the reach of the medical and research community in an effort to provide evidence-based answers to the complex issues that families face.

### Strengths and Limitations

This growing community to support families affected by HD demonstrates a level of interaction that suggests satisfaction and commitment of users, an interrelationship that has an effect on sustainment of a virtual community [24]. The campaign has been designed and marketed to catch attention and resonate with members to ensure viability and sustainability.

Limitations of this study include the retrospective nature of our analysis. As this is a parent-initiated and parent-led campaign, it was not designed at inception to be scientifically evaluated. Internet analytics however, in combination with the vigilant management of this community have provided detailed quantitative and qualitative data on a real-world campaign dedicated to a health issue. Second, although a geographically wide representation has been achieved, this study includes caregivers of children with HD who have Internet access, the knowledge and skills to access online forums, and the desire to participate in one of the three online forums. Results specifically pertaining to the content analysis may not be representative of the overall caregiver community. Finally, this study was not designed to assess the value of the HD Community to its members. Further research is needed to understand the effect of these types of campaigns on individuals who view and actively participate in the conversations (active users) as well as those who view information but do not personally contribute (passive users).

### Conclusions

Social media has changed the way people communicate. This study demonstrates that by using a multipronged social media strategy, one has the ability to break through barriers of distance (global) and time (instant publication) while targeting the desired demographic. These characteristics make social media an effective tool to build a community as well as collect and disseminate information. The Hirschsprung’s community has been connected through the Shit Happens Campaign. An HD Twitter account, Facebook page, and a personal blog dedicated to supporting families living with HD have been viewed hundreds of thousands of times across the globe. The sites are presently filled with questions from families who continue to struggle to manage even after surgical intervention for HD, presenting health care professionals and researchers with the opportunity to provide families with evidence-based information to guide care. Families rely on these forums for support from other caregivers, but clinicians and researchers are not represented within these social media communities. Partnerships between communities of caregivers, health clinicians, and researchers mediated through social media could provide unprecedented opportunity for consumer-driven research. Together, families and care providers can ensure that caregiver concerns become research priorities and that existing evidence and research results are widely and appropriately shared.
